# A parallel adaptive quantum genetic algorithm for the controllability of arbitrary networks

**DOI:** 10.1371/journal.pone.0193827

**Published:** 2018-03-19

**Authors:** Yuhong Li, Guanghong Gong, Ni Li

**Affiliations:** 1 School of Automation Science and Electrical Engineering, Beihang University, Beijing, China; 2 State Key Laboratory of Virtual Reality Technology and Systems, Beihang University, Beijing, China; Universidad Rey Juan Carlos, SPAIN

## Abstract

In this paper, we propose a novel algorithm—parallel adaptive quantum genetic algorithm—which can rapidly determine the minimum control nodes of arbitrary networks with both control nodes and state nodes. The corresponding network can be fully controlled with the obtained control scheme. We transformed the network controllability issue into a combinational optimization problem based on the Popov-Belevitch-Hautus rank condition. A set of canonical networks and a list of real-world networks were experimented. Comparison results demonstrated that the algorithm was more ideal to optimize the controllability of networks, especially those larger-size networks. We demonstrated subsequently that there were links between the optimal control nodes and some network statistical characteristics. The proposed algorithm provides an effective approach to improve the controllability optimization of large networks or even extra-large networks with hundreds of thousands nodes.

## Introduction

The real world consists of ubiquitous intricate and intertwined networks. Some are tangible, such as traffic networks [1, 2], power networks [3], and financial networks [4], whereas others are invisible networks that penetrate into every aspect of our lives, such as interpersonal relationship networks [5, 6], wireless networks [7, 8], and ecological networks [9]. The expected goal of research into a complex network is to be able to regulate and control it from the outside, achieve the desirable state or performance by injecting outward control signals to some network nodes (called driver nodes), and ultimately achieve the real controllability of a complex network.

Many studies have been conducted on the relationship between network topology and network controllability [10–13]. Researchers have proposed that all hub nodes with a high degree or betweenness centrality could be chosen as driver nodes [14]. Jalili et al. [15] found that an optimum driver node could not always be a hub node. To elucidate the configuration of driver nodes for the optimum network pinning control, a differential evolution method was used. The method worked well, but it was only suitable for undirected networks. Assuming that the objective network had finite-dimensional linear dynamics [16], the network could be structurally controlled by applying one time-varying input to the power dominating set. In the practical applications with economical and physical constraints, driver nodes can not always be freely selected to be injected to network nodes. In this context, Lo et al. [17] addressed a geometrical framework for the partial controllability issue of networks by solving an integer linear programme. The approach was also suitable to optimize the complete controllability of networks. The network permeability index provided a quantitative understanding of the challenge of controlling a network partially or completely.

The dynamics of the network could be expressed as a linear time-invariant system $\dot{x(t})=Ax(t)+Bu(t)$, where x(t) is the state vector of the network. Assuming a network has *N* state nodes and P control nodes, Aϵ*R*^*N*×*N*^ is the coupled matrix between state nodes, u(t) is the control or input vector forced on the network, and Bϵ*R*^*N*×*P*^ is the input matrix. The general approach for the controllability problem $\dot{x(t})=Ax(t)+Bu(t)$ is to determine a proper input matrix based on the Kalman rank condition such that the pair (A, B) is controllable [10]. However, this controllability problem has a large computational load with 2^*N*^ possibilities assuming each node can be either driven or not driven [11], and this exponential growth is especially rapid when the network size is large. To overcome this difficulty, Liu et al. [10] introduced the structural controllability concept [18], which ensured that the Kalman rank condition was verified. They first found that the number of driver nodes for the full controllability of a complex network mainly depended on the network degree distribution. The process controllability of network dynamics was explored by transforming node dynamics into edge switch dynamics [19] and resulted in similar controllability conclusions to those obtained by Liu et al. [10].

The structural controllability methods based on graphical analysis of pair (A, B) for the system $\dot{x(t})=Ax(t)+Bu(t)$ [18] could identify n_D_ for arbitrary directed networks [10]. Several effective methods have been proposed to identify the minimum number of driver (control) nodes (n_D_), for example, the maximum matching (MM) method [20], the cavity method [21], and an extremal optimization (EO) algorithm [22]. The computational load of determining n_D_ could be effectively reduced based on the MM method, which has the computational complexity of $O(\sqrt{N}L)$, where L is the number of linked edges between state nodes. EO [22] was proposed based on the Kalman rank condition to identify n_D_ for the full controllability of directed networks with the computational complexity of O(N^4^*P*^3^). However, this structural controllability framework [10] is not applicable to undirected networks for the symmetric characteristic of the network matrix or networks with exact link weights [11, 23, 24]. These limitations prompt the development of exact network controllability theory, which is an exact controllability framework for the controllability of complex networks with arbitrary network structures and link weights. It optimizes the complete controllability of networks based on the Popov–Belevitch–Hautus (PBH) rank condition [25], which is an alternative criterion that is equivalent to the Kalman rank condition [26]. The PBH controllability method requires the sequential computation of the eigenvalues of the N × N matrix A and the rank of the N × (N + P) PBH matrix. The computational complexity of the eigenvalue computation of matrix A and the PBH matrix rank is O(*N*^3^), O((*N* + *P*)^3^), respectively [27]. Thus, the computational complexity of the PBH method is O((*N* + *P*)^3^). The Kalman controllability method does not require an eigenvalue computation. However, it requires the rank computation of the N × NP Kalman matrix with the computational complexity of O(*N*^3^*P*^3^), which is larger than that of the PBH controllability method. Representative exact structural controllability methods consist of a maximum multiplicity theory (MMT) [11] and an effective self-adaptive genetic algorithm (GA) [28]. n_D_ was computed based on the MMT to be equal to the maximum geometric multiplicity of all eigenvalues of the network [11], and the computational complexity is O(*N*^2^(logN)^2^). The GA [28] was studied to identify n_D_ of arbitrary networks, and the computational complexity is O(2m × (*N* + *P*)^3^ × *l*), where 2m is the population size and *l* is the number of different eigenvalues of the controlled network.

The authors demonstrated the evolution process of network topology of two networks (Erdős–Rényi (ER) and scale free (SF)) [28], i.e., the dynamic change of network topology with different number of control nodes being injected to network state nodes. The GA algorithm [28] exceeded the EO [22] much in terms of the convergence speed and iterations. However, networks with the more complexity than the scale 150 were not studied in [28] and the maximum network scale that EO processed was 200 [22]. And the convergence speed and iterations were still not satisfactory, for example, for ER, with both 150 state nodes and 150 control nodes, and the average degree 5.0, n_D_ converged at the 101st generation after 398.03 s [28]. The results showed that it remained a challenge for the two algorithms to optimize networks with hundreds of thousands or even larger networks. Additionally, almost all real networks have small-world (SW) properties with a large cluster coefficient and short average distance [29] (e.g., power grids, transportation networks, and social networks). The addition of the SW network controllability study is also significant for better mimicking reality.

Therefore, based on the PBH rank condition, we propose a parallel quantum genetic algorithm (PAQGA) to more rapidly determine the minimum number of control nodes. The proposed algorithm is suitable for arbitrary networks that comprise both control nodes and state nodes. The simulation results for a series of benchmark networks demonstrate the effectiveness of the algorithm. Furthermore, we demonstrate the relationship between the controllability of a network and its network properties such as network average degree, degree heterogeneity, power-law index, and clustering coefficient.

The remainder of this paper is organized as follows: In Section 2, we provide a description of the issue in which a network can be controlled through a small amount of control nodes. In Section 3, we introduce the PAQGA for the solution of the minimum number of control nodes to exactly control arbitrary networks. In Section 4, we analyze and discuss the performance and experimental results of the proposed framework by studying popular ER, SW, SF, and some real-life networks. We draw conclusions and suggest future work in Section 5.

## Problem definition

In this paper, we provide a descriptive definition of the entire controllability problem of a directed weighted network.

**Fig 1:**
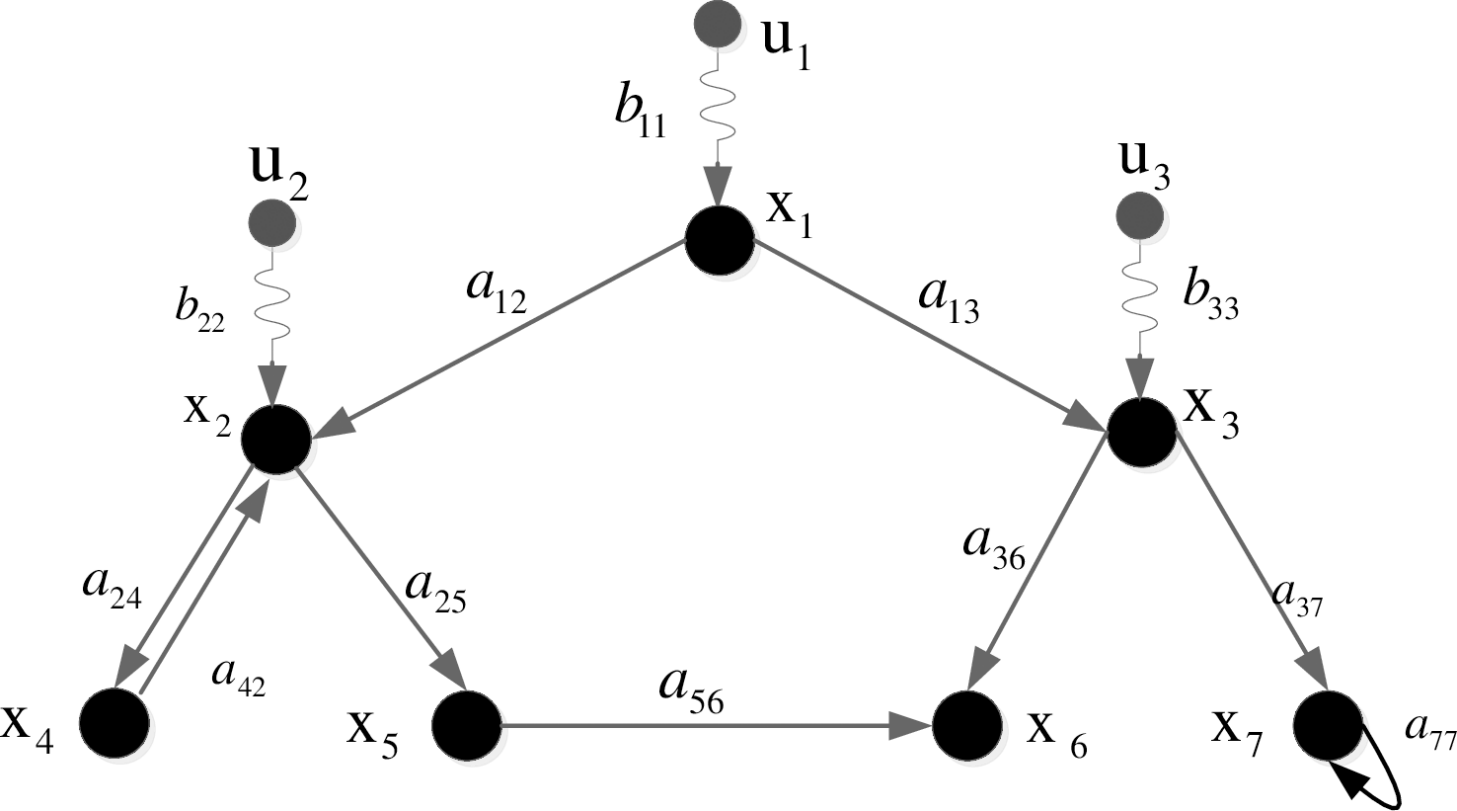
Example of a directed network, where V_s_ = {x_1_,x_2_,x_3_,x_4_,x_5_,x_6_,x_7_}, V_c_ = {u_1_,u_2_,u_3_}, E_s_ = {(x_1_,x_2_),(x_1_,x_3_),(x_2_,x_4_),(x_2_,x_5_),(x_3_,x_6_),(x_3_,x_7_),(x_4_,x_2_),(x_5_,x_6_),(x_7_,x_7_)}, E_c_ = {(u_1_, x_1_),(u_2_, x_2_),(u_3_, x_3_)}, a_ij_ (i = 1,2,…,7; j = 1,2,…,7) ∈ V_s_ × V_s_, b_ij_(i = 1,2,3; j = 1,2,…,7) ∈ V_c_ × V_s._, *w*_24_ > 0 and *w*_42_ > 0.

**Definition 2.1** [22]. A network that contains P control nodes and N state nodes can be expressed as a triple tuple G = (V,E,W), where V = *V*_*s*_ ∪ *V*_*c*_, *V*_*s*_ = {*v*_1_,*v*_2_,…,*v*_*N*_} = {*x*_1_,*x*_2_,…,*x*_*N*_} is the set of state nodes, and *V*_*c*_ = {*v*_*N+*1_,*v*_*N+*2_,…,*v*_*N+P*_} = {*u*_1_,*u*_2_,…,*u*_*P*_} is the set of control nodes; E = *E*_*s*_ ∪ *E*_*c*_, *E*_*s*_ ∈ *V*_*s*_ × *V*_*s*_ is the set of the linked edges between state nodes, and *E*_*c*_ ∈ *V*_*c*_ × *V*_*s*_ is the set of linked edges between control nodes and state nodes, where each state node can only be connected to one control node; and W ∈ *R*^(*N* + *P*) × (*N* + *P*)^ is the set of edge weights, *w*_*ij*_ = 0 if there is not a link between *v*_*i*_ and *v*_*j*_; otherwise, *w*_*ij*_ (*w*_*ji*_) represents the strength that *v*_*i*_ (*v*_*j*_) could affect *v*_*j*_ (*v*_*i*_), *w*_*ij*_ (*w*_*ji*_) > 0 if the direction is i → j (j → i). Fig 1 shows an illustration of the definition.

**Remark 2.1** [22]. The set W can be expressed by the representation of a block matrix that contains *A* and B as follows:

$$W=\left[\begin{array}{cc} A & B\\ 0 & 0 \end{array}\right],$$ (1)

**Fig 2:**
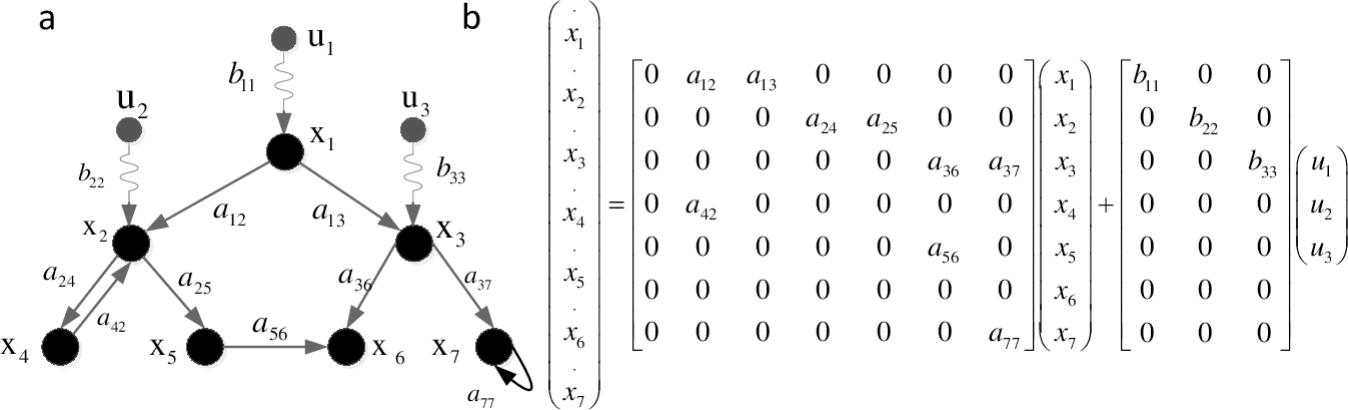
Corresponding dynamics equation of the network in Fig 1, where x = {x_1_, x_2_, x_3_,x_4_,x_5_,x_6_,x_7_}, u = {u_1_,u_2_,u_3_}, a_ij_ (i = 1,2,…,7; j = 1,2,…,7) is the connection weight from state node i to state node j, and b_ij_(i = 1,2,3; j = 1,2,…,7) is the connection weight from control node i to state node j.

**Definition 2.2** [10, 22]. The network G = (V,E,W) can be represented by

$$\dot{x(t})=Ax(t)+Bu(t),$$ (2)

where **x**(t) = (*x*_1_(*t*),*x*_2_(*t*),…,*x*_*N*_(*t*))^*T*^ is the state vector of the network, Aϵ*R*^*N*×*N*^ is the coupled matrix between state nodes, u(t) = (*u*_1_(*t*),*u*_2_(*t*),…,*u*_*P*_(*t*))^*T*^ is the control or input vector forced on the network, Bϵ*R*^*N*×*P*^ is the input matrix, and B = {*b*_*ij*_}, *b*_*ij*_ is the weight of a directed link that the input signal *u*_*j*_(j = 1,2,…,P) points to the network state node *x*_*i*_ (*i* = 1,2,…,*N*). For simplicity, hereafter the time symbol (t) will be omitted. Fig 2 shows the equation expression of the network in Fig 1.

**Definition 2.3** [22]. A control scheme D of a network G = (V,E,W) is determined by the selected control nodes with definite number and their acting position. D could be represented by a binary diagonal matrix as D = diag{*d*_1_,*d*_2_,…,*d*_*P*_}, where *d*_*i*_(*i* = 1,2,…,*P*) is a variable of value zero or one, and *d*_*i*_ = 1 means that the control node *u*_*i*_ is chosen to be a component of the network control strategy; otherwise, *u*_*i*_ is removed together with its associated links.

**Remark 2.2** [22]. Based on Definition 2.3, a novel control scheme D* is determined for which a different set of control nodes is selected. Then a novel network topology is generated as G* = (V*,E*,W*), where V* ∈ *V*, E* ∈ *E*, and W* ∈ *R*^(*N* + *r*) × (*N* + *r*)^, where r is the number of selected control nodes. Accordingly, the network dynamics are also changed as

$$\dot{x}=Ax+B^{*}u^{*},$$ (3)

where B* ∈ *R*^*N*×*r*^ is the new input matrix that represents the connections between new chosen control nodes and network state nodes, and u* ∈ *R*^*r*^ is a time-variable input vector that contains r control nodes.

**Definition 2.4** [22]. Mϵ*R*^*P*×*r*^ is the index set of the selected control nodes, M = {*m*_*ij*_}, and *m*_*ij*_ = 1 means that the *j*_*th*_ chosen control node is *u*_*i*_, *i* = 1,2,…,P, j = 1,2,…,r, r ≤ P.

**Remark 2.3** [22]. M is constructed by the nonzero columns of the control scheme D*. For example, if u* = {*u*_1_,*u*_2_,*u*_3_} is chosen from a previous control node set *u* = {*u*_1_,*u*_2_,*u*_3_, *u*_4_} to be a new control scheme D* = *diag*{1,1,1,0}, then M is obtained from this D* as

$$M=\left[\begin{array}{ccc} 1 & 0 & 0\\ 0 & 1 & 0\\ 0 & 0 & 1\\ 0 & 0 & 0 \end{array}\right],$$ (4)

**Remark 2.4** [22]. The evolving input matrix and input vector can be revised as B* = BD*M and u* = M^T^*u*, respectively. Then Eq (3) can be rewritten as

$$\dot{x}=Ax+BD^{*}M(M^{T}u),$$ (5)

**Fig 3:**
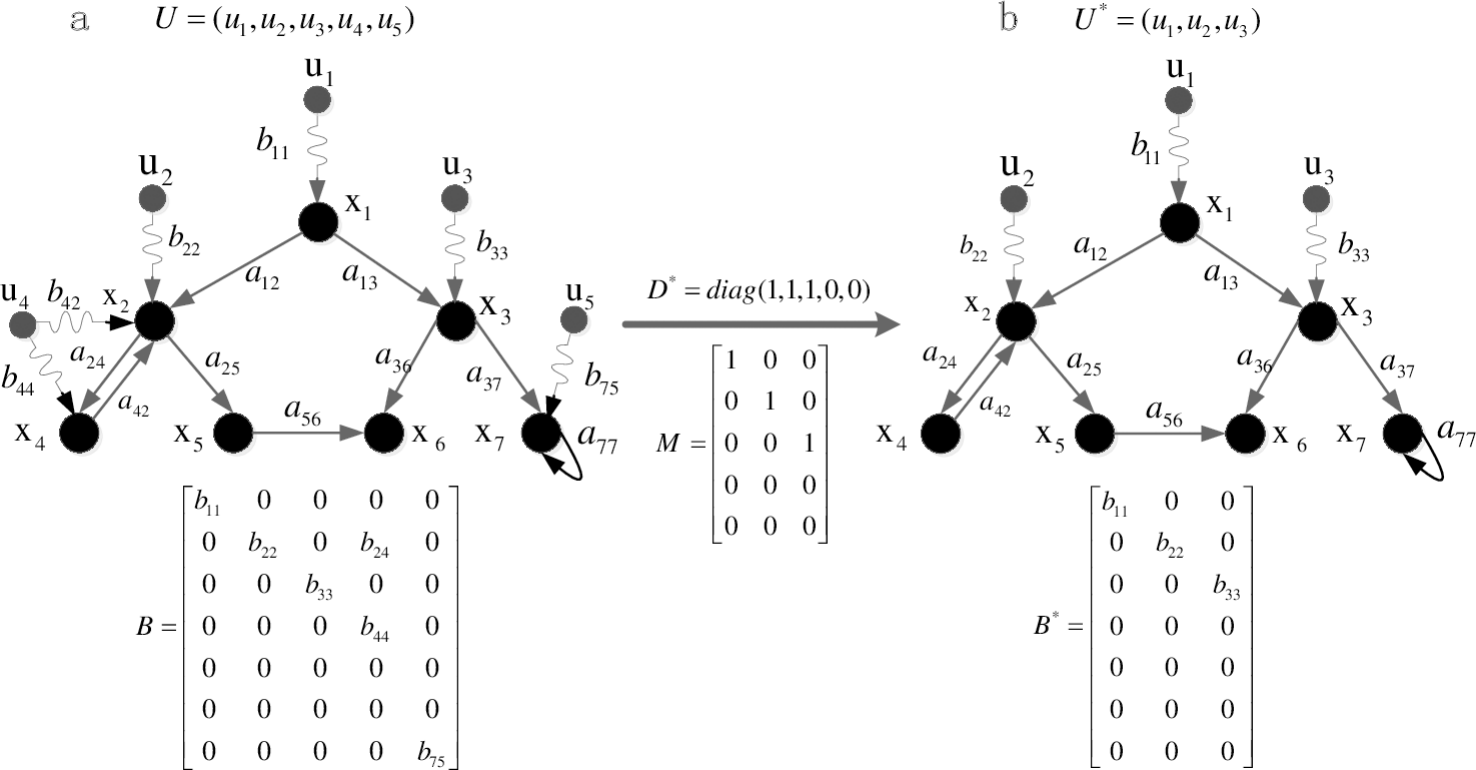
Illustration of how new control scheme D* functions in the network topology. (a) Original network with seven state nodes and five candidate control nodes. (b) Input matrix changes into B* = BD*M after choosing u* = {u_1_,u_2_,u_3_} as new control nodes. New network has seven state nodes and three control nodes.

Fig 3 shows a simple case that illustrates how a control scheme influences the network topology.

**Remark 2.5.** Based on the PBH rank condition [25], the network G = (V,E,W) (Eq. (2)) can be steered to any desired state within a finite time, that is, G is fully controllable if and only if

$$rank(\lambda_{i}I_{N}-A,B)-N=0,$$ (6)

and the new system (Eq. (5)) is fully controlled if and only if

$$rank(\lambda_{i}I_{N}-A,BD^{*}M)-N=0,$$ (7)

is satisfied for each different eigenvalue *λ*_*i*_ of the state matrix A, where *I*_*N*_ ∈ *R*^*N*×*N*^ is an identity matrix.

For an arbitrary network G = (V,E,W), our purpose is to determine the minimum control nodes to guarantee its full control. Based on the above analysis, the controllability problem can be transformed into a single target restricted optimization problem as

$$\min_{D}\sum_{j=1}^{P}d_{j},$$ (8)

subject to

$$rank(\lambda_{i}I_{N}-A,BDM)-N=0,{\forall \lambda }_{i}\in eig(A),$$ (9)

$$d_{j}=\{0,1\},j=1,2,\ldots ,P,$$ (10)

where *A* is the state matrix, *B* is the original input matrix, *D* is the original control scheme, *M* is the indicator matrix that is derived from the nonzero column of *D*, N and *P* are the dimensions of *A* and *B*, respectively, *λ*_*i*_ is the eigenvalue that belongs to *A*, *eig*(*A*) is the set of different eigenvalues of *A*, *d*_*j*_ is the element of *D*, and *d*_*j*_ = 1 when *u*_*j*_ is selected and *d*_*j*_ = 0 otherwise.

## Solution framework

### Overview of the QGA

GA is a global optimization method that can optimize problems with multiple parameters to reach near the global optima [30–33]. However, in some practical applications, it often requires multiple iterations because of the slow convergence speed and prematurity features, and easily falls into the local minima [34, 35]. Additionally, for many complex problems, a large population is required to obtain the optimal solution. The convergence of GA mainly depends on the selecting operation, which largely affects the convergence speed. Additionally, its searching capability mainly relies on crossover and mutation operations, which primarily affect the occurrence of the premature phenomenon. Therefore, regarding enhancing GA search performance, the approach used to choose suitable selecting, crossover, and mutation strategies has been always an urgent and pivotal issue in the study and application of GA [33, 36].

For small and medium-sized applications, the solution could be achieved within a tolerance range using GA. However, a gene (typically encoded with a 0–1 string) in a GA chromosome typically delivers certain information, which limits the population diversity. It performs worse in multivariate issues, for example, the controllability study of complex networks, which mostly has complex structures, and large-size nodes and links.

Combining quantum computing and GA, and adopting qubits as the representation of chromosome genes [37], QGA is a proper intelligent optimization algorithm for solving the network controllability problem [38]. These QGA qubits cover all possibilities for the linear superposition property of quantum information, which could reduce the algorithm’s complexity and promote the achievement of the optimal solution under a smaller population [37].

In quantum computing, |0⟩ and |1⟩ signify two basic states of microscopic particles. According to the principle of the superposition property, the superposition state of quantum information could be the linear combination of the two basic states [39], which can be written as

$$|\varphi \rangle =\alpha |0\rangle +\beta |1\rangle ,\;{\left|\alpha \right|}^{2}+{\left|\beta \right|}^{2}=1,$$ (11)

where α and β are the state probability amplitudes of a qubit, and α^2^ and *β*^2^ are the probability that a qubit changes to be state |0⟩ and state |1⟩, respectively. One qubit also can be expressed as $\left[\begin{array}{c} \alpha \\ \beta \end{array}\right]$.

Assume the number of optimization variables is n and the population size is 2m. The i_th_ chromosome is denoted by G_i_(i = 1,2,…,m) as

Gi=[αi1αi2…αinβi1βi2βin], (12)

where $\alpha_{i1}^{2}+\alpha_{i2}^{2}+\beta_{i1}^{2}+\beta_{i2}^{2}=1,i=1,2,\ldots ,m$. G_i_ contains two parallel gene chains or individuals (α_i1_,α_i2_,…,α_in_ and β_i1_,β_i2_,…,β_in_). Each individual is a candidate solution of an optimization problem:

$$G_{i}=\left[\begin{array}{c} G_{i1}\\ G_{i2} \end{array}\right],\;\{\begin{array}{c} G_{i1}=\left[\alpha_{i1},\;\alpha_{i2},..,\alpha_{\mathrm{ij}},..,\alpha_{\mathrm{in}}\right]\\ G_{i2}=\left[\beta_{i1},\;\beta_{i2},\ldots ,\beta_{\mathrm{ij}},..,\beta_{\mathrm{in}}\right] \end{array},$$ (13)

QGA and enhanced QGA have already been studied to optimize many combinational problems [40–42]. For example, QGA overmatches classic GA with less complexity and higher performance in 0–1 combinational optimization problems [39]. Adaptive QGA models were proposed and tested on classical combinational problems, such as knapsack, maxcut and onemax [38], the multi-aircraft cooperative target allocation problem, and constrained engineering design problems [43]. However, the time efficiency was not seriously stressed. To increase the speed, a parallel QGA was developed and effectively applied to a knapsack problem [44]. It divided the entire population into subpopulations on different parallel processors and used the migration rate for the information exchange of these subpopulations. However, the Q-gate rotation was implemented according to a fixed lookup table, which did not take full advantage of the dynamic differences between individuals during the iterating process.

Inspired by current achievements, to quickly and efficiently solve the controllability problem of complex networks, we investigated a PAQGA scheme, in which: 1) partial programs of the algorithm are executed in parallel; 2) a set of adaptive Q-gate rotation rules are proposed and adaptive crossover operation are used; and 3) population catastrophe is implemented to accelerate convergence.

### Workflow of the PAQGA for network controllability

**Fig 4:**
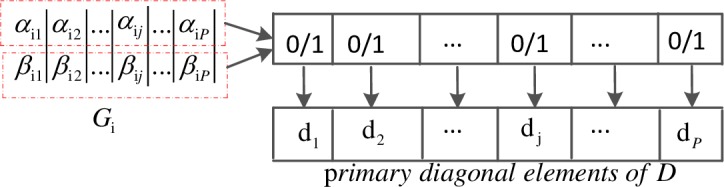
Chromosome encoding in quantum genetic algorithm.

Based on the above, each control scheme *D* is a diagonal matrix, whose elements on the primary diagonal are either zero or one. Therefore, we adopt the binary mechanism to encode the algorithm chromosome *G*_*i*_(*i* = 1,2,…,*m*), and each binary gene chain can represent a control scheme, as shown in Fig 4.

To apply PAQGA conveniently, a penalty term *Pen*_*i*_(*D*) is defined to convert the optimization problem described in Eqs (8), (9) and (10) into an unconstrained optimization problem. *Pen*_*i*_(*D*) is used to evaluate the *i*_*th*_ perturbation for a specific control scheme *D*:

$$Pen_{i}\left(D\right)=\sigma_{i}\times {\left(rank\left(\lambda_{i}I_{N}-A,BDM\right)-N\right)}^{2},i=1,2,\ldots ,l,$$ (14)

where *σ*_*i*_ is the penalty coefficient defined as *σ*_*i*_ = {*cσ*_*i*−1_} (*σ*_1_ = 10*P*, *c* > 0) is a strictly increasing positive sequence to reduce the calculation burden of minimizing the penalty function, and *l* is the total number of distinct eigenvalues of *A*. The overall penalty of *D* is the sum of *Pen*_*i*_(*D*) expressed as

$$Pen(D)=\sum_{i=1}^{l}{Pen}_{i}(D),$$ (15)

According to the PBH rank condition, when the network G = (V,E,W) is fully controllable, *Pen*(*D*) should be zero and vice versa. Therefore, the fitness function can be achieved by merging the penalty term into the optimization Eqs (8), (9) and (10):

$$\begin{array}{l} f(D)=\sum_{j=1}^{P}d_{j}+Pen(D)\\ \;=\sum_{j=1}^{P}d_{j}+\sum_{i=1}^{l}\sigma_{i}\times {\left(rank(\lambda_{i}I_{N}-A,BDM)-N\right)}^{2}, \end{array}$$ (16)

**Fig 5:**
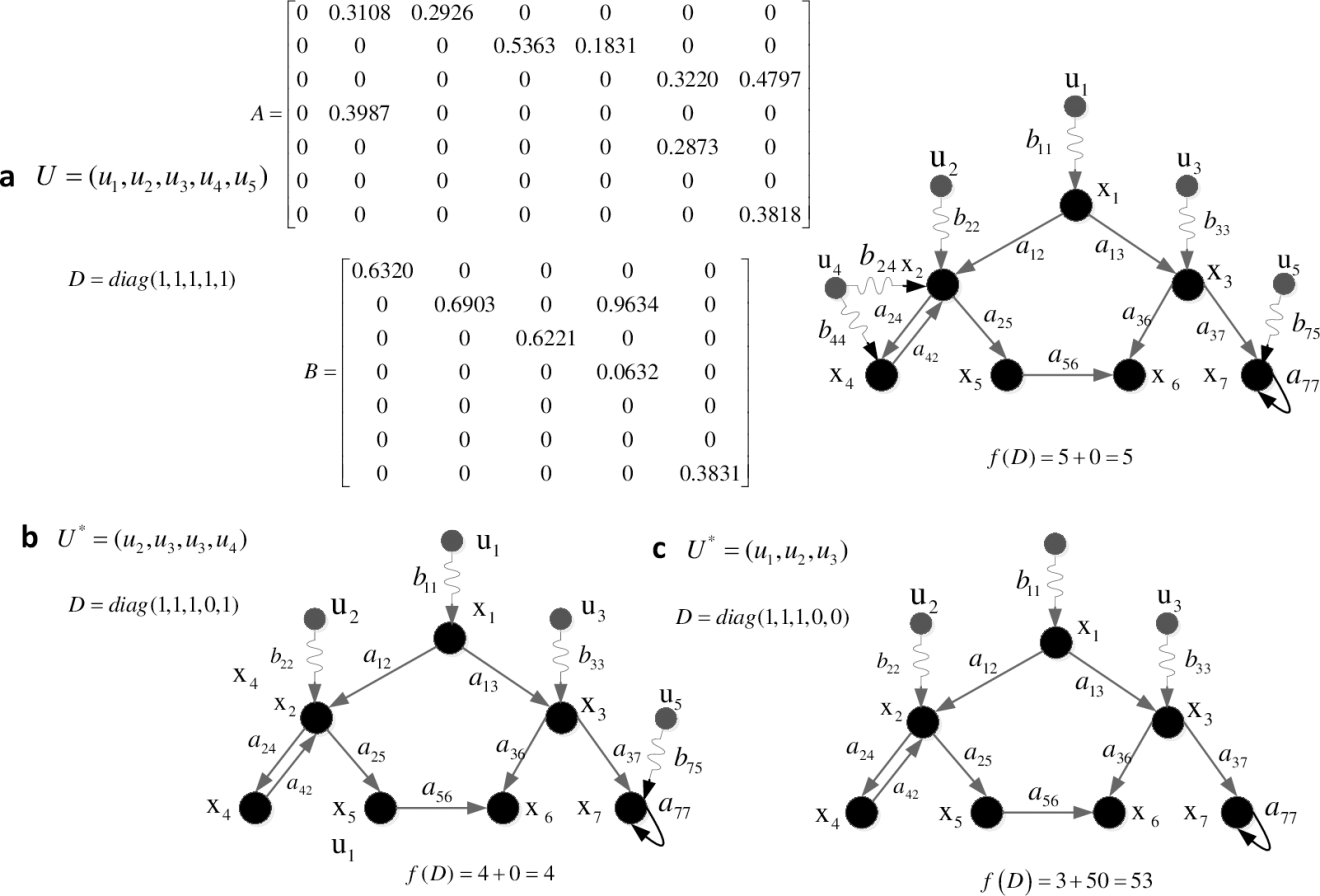
Illustration of the fitness evaluation for different control schemes on a directed weighted network with self-loop. (a) Initial network with seven state nodes and five control nodes. Connecting weights are randomly assigned between zero and one. Fitness of initial D = diag{1,1,1,1,1} is f(D) = 5 and the penalty term is zero; thus, the network is entirely controllable. (b) When D changes to new D = diag{1,1,1,0,1}, f(D) is four, the penalty is zero, and the network is still fully controllable. (c) u_4_ is removed from (b). For simplicity, *c* is set to 1, and the penalty term $pen(D)=10P*\sum_{i=1}^{l}{Pen}_{i}(D)=50*1\neq 0$ indicates that the network with this topology cannot be fully controlled.

For the optimization problem, our objective is to minimize f(D) based on the 0–1 integer values of *d*_*j*_ (*j* = 1,2,…,*P*). Fig 5 shows the fitness evaluation of different control schemes on a simple network.

**Fig 6:**
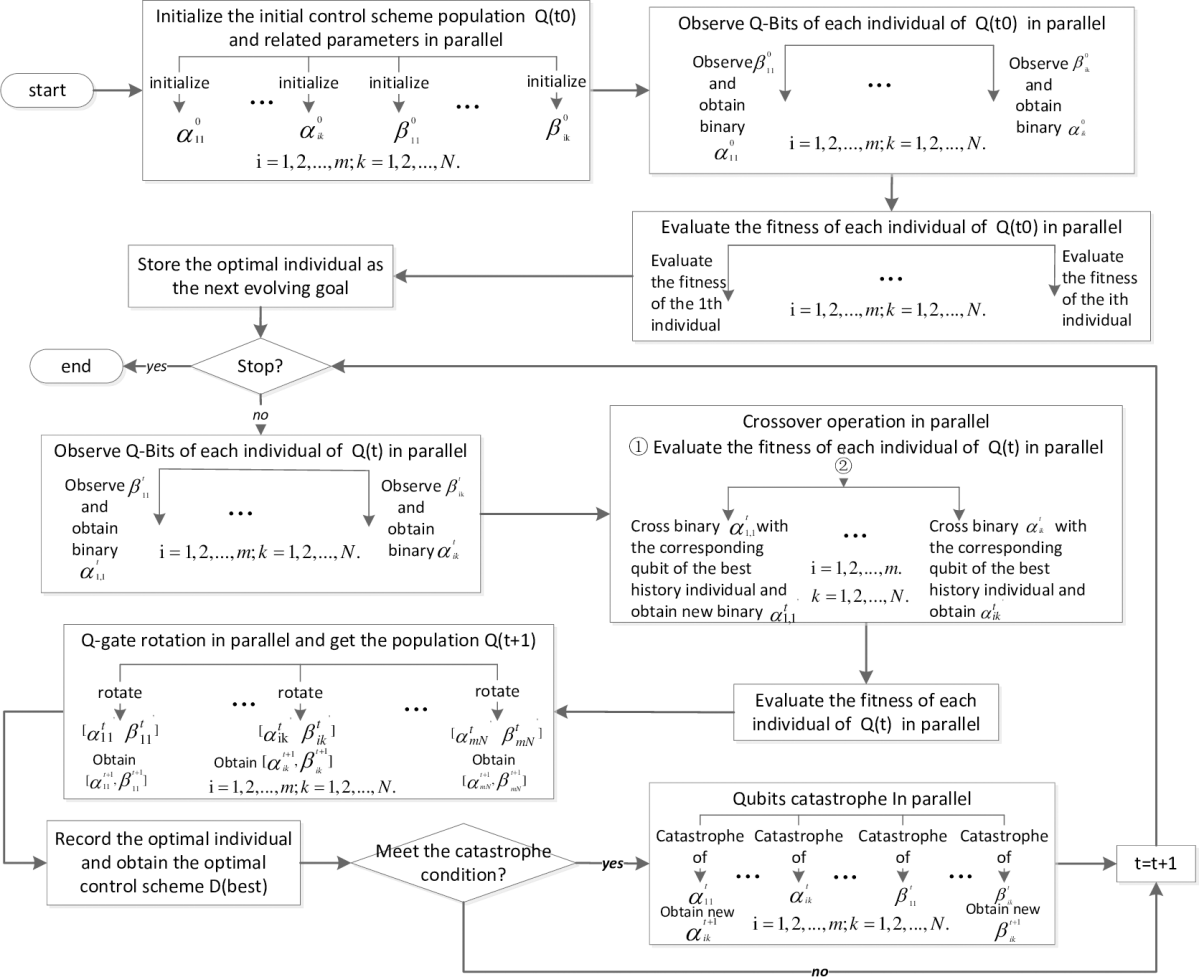
Flow chart of the PAQGA.

After defining the chromosome representation and fitness function, the network controllability problem can be optimized using the following steps. Fig 6 shows the flow chart of the proposed PAQGA for the optimization problem.

**Step 1:** The population at the t_*th*_ generation is denoted as $Q(t)=\{G_{1}^{t}{,G}_{2}^{t},\ldots ,G_{m}^{t}\},$

Git=[αi1tαi2t…αiPtβi1tβi2tβiPt],i=1,2,…,m,t=0,2,…,maxgen−1, (17)

where N is the number of qubits, that is, the number of network state nodes, and *maxgen* is the maximum iterating generation.

Initialize the initial population as

$$Q(t_{0})=\{G_{1}^{0}{,G}_{2}^{0}{,\ldots ,G}_{m}^{0}\},$$ (18)

where Gi0=[αi10αi20…αiP0βi10βi20βiP0],i=1,2,…,m.

All the quantum states (α_ik_ and β_ik_) in the PAQGA are initialized in parallel with the value $\frac{1}{\sqrt{2}}$, i = 1,2,…,m, k = 1,2,…,N. Additionally, set *D*_*best*_ = *D*_0_, the iterative generation t = 1, and *σ*_1_ = 10*P*.

When we set the initial control scheme D_0_ = *diag*{*d*_*j*_ = 1},*j* = 1,2,…,*P*, the initial best fitness value is f(D_*best*_) = *P* + 0 = *P*. It is easily proved that with *σ*_*i*_ = {*cσ*_*i*−1_} (*σ*_1_ = 10*P*, *c* > 0), the fitness f(D) ≤ *P* if and only if the control scheme D always satisfies the constraint Eq (9). With the initialization f(D_*best*_) = *P*, whenever D_*best*_ is updated by *D*_*i*_, we have f(*D*_*i*_) < f(D_*best*_) = *P*, which means *D*_*i*_ meets the constraint Eq (9). Thus, D_*best*_ always evolves in the feasible region that makes the network entirely controllable.

**Step 2:** Observe the qubits of each individual of Q(*t*_0_) in parallel following the rules in Section 3.3 and obtain the binary strings.

**Step 3:** Evaluate each individual of Q(*t*_0_) in parallel and save the optimal individual as the evolving goal in the next generation.

**Step 4**:

While (*maxgen* is not reached), do the following:

(a) Observe the qubit value of each individual of Q(t) in parallel following the rules in Section 3.3.
(b) To increase the diversity of the population and inherit the excellent genes from the previous population, the adaptive crossover operation is performed in parallel in accordance with Section 3.4.
(c) Evaluate each individual of Q(t) in parallel and store the optimal individual as the evolving goal in the next generation.
(d) Perform the Q-gate rotation operation in parallel and obtain the offspring population Q (t+1).

For each individual, two parallel gene chains update simultaneously. Rotation angle θ is first computed based on Section 3.5 and then the qubits are updated by Q-gate rotation. The Q-gate is expressed as [45]

$$Q\text{-}gate=\left[\begin{array}{cc} cos\theta & -sin\theta \\ sin\theta & \;cos\theta \end{array}\right],$$ (19)

The Q-gate rotation operation is

$$\left\{ \begin{array}{c} \alpha_{ik}^{t+1}=cos\theta *{\alpha_{ik}^{t}}^{\prime }-sin\theta *{\beta_{ik}^{t}}^{\prime }\\ \beta_{ik}^{t+1}=sin\theta *{\alpha_{ik}^{t}}^{\prime }+cos\theta *{\beta_{ik}^{t}}^{\prime } \end{array}\right.$$ (20)

where *i* = 1,2,…,*m*, k = 1,2,…,*N*, *t* = 1,2,…,*maxgen* − 1, and θ = δ * s, where δ is the rotation angle value and s is its sign.

(e) Record *D*_*best*_ and f(D_*best*_).
(f) If the optimal values of the past several successive generations are the same, then perform the parallel population catastrophe operation.

The fitness function evaluation and the crossover operation are the two most time-consuming steps in the process of the flow execution. Assume that η parallel processors are used, the cost is $O(\frac{m\times {\left(N+p\right)}^{3}\times l}{\eta })$ and $O(\frac{m\times {\left(N+p\right)}^{3}\times l}{\eta^{2}})$, respectively, where *l* is the number of different eigenvalues of the controlled network. Therefore, the computational complexity of the PAQGA is $O\left(\frac{m\times {(N+p)}^{3}\times l}{\eta }\right)$.

### Observing operation

Each qubit of the chromosome can be adjusted to be at a stationary state using an observation operation. We adopt a random observing method by running the following pseudocode in parallel:

start

            if$(rank(k)\geq {\left(\beta_{ik}^{t}\right)}^{2}),i=1,2,\ldots ,m;k=1,2,\ldots ,N$

                        return binary $\alpha_{ik}^{t}=1$;

            else

                        return binary $\alpha_{ik}^{t}=0$;

end

where rand(k) is a random digit. If rand(k) is not less than ${\left(\beta_{ik}^{t}\right)}^{2}$ (the probability to be state |1⟩), then the observed value of the qubit $\alpha_{ik}^{t}$ is 1 and 0 otherwise.

### Crossover operation

**Fig 7:**
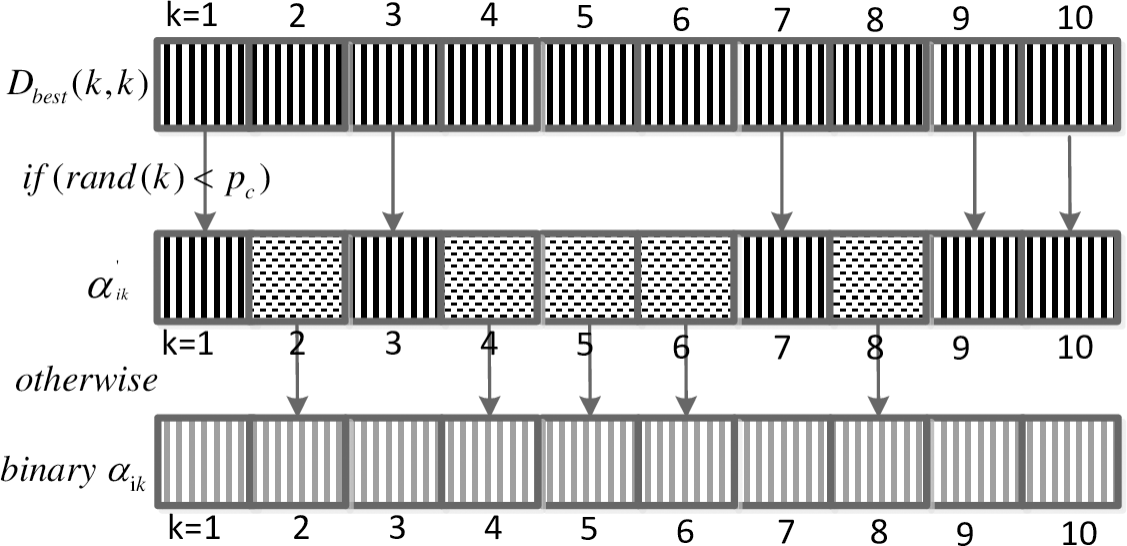
Simple crossover example.

The crossover operator is an important operation of GA. Information about individuals can be exchanged using the operation. Subsequently, excellent genes could be reserved for population evolution to move in a better direction. To increase the diversity of the population and improve the optimization performance of PAQGA, the crossover operator is introduced. We obtain novel binary values $\alpha_{ik}^{\prime }$ by crossing each binary qubit value α_*ik*_,i = 1,2,…,m, k = 1,2,…N with corresponding information on the historically best control scheme, that is, *D*_*best*_(*k*,*k*), based on a certain crossover probability. The specific crossover mode is

$$\alpha_{ik}^{\prime }=\left\{ \begin{array}{c} D_{best}(k,k),\;if\;rand(k)<p_{c}\\ \alpha_{ik},\;otherwise,\;i=1,2,\ldots ,m;k=1,2,\ldots ,N, \end{array}\right.$$ (21)

where *i* is the *i*_*th*_ individual, *rand*(*k*) is a random number between [0, 1], and *p*_*c*_ is the crossover probability. Fig 7 shows a simple crossover example with 10 qubits to explain the rule (21).

In the early days of population evolution, there existed relatively big differences between individuals. Therefore, the crossover possibility to produce better offspring should have been bigger. Moreover, if we increased the crossover probability at this time, the evolution process would have been accelerated. By contrast, in the late stages of evolution, differences between individuals became smaller as the best solution was approaching. The crossover probability should have been correspondingly diminished to reserve the good genes. We design an adaptive crossover operator as

$$p_{c}\left(i\right)=\{\begin{array}{c} \frac{m}{Q_{i}}p_{c0}\exp\left(-\frac{\left|f_{max}-f\left(G_{i}\right)\right|}{f_{max}-f_{min}}\right),\;f_{max}\neq f_{min}\\ \frac{m}{Q_{i}}p_{c0},\;f_{max}=f_{min} \end{array},\;i=1,2,\ldots ,m,$$ (22)

where *p*_*c*_(*i*) is the crossover probability of the *i*_*th*_ current individual, *Q*_*i*_ is the number of those individuals whose fitness is better than that of the historically best individual, *p*_*c*0_ is the initial crossover probability, *f*_*max*_ and *f*_*min*_ are the previous worst fitness and best fitness, respectively, and *f*(*G*_*i*_) is the fitness of the *i*_*th*_ current individual.

We can observe that *p*_*c*_(*i*) becomes bigger when the control scheme *G*_*i*_ becomes worse and vice versa. Moreover, *p*_*c*_(*i*) is inversely proportional to *Q*_*i*_, which means that if there are not so many good individuals, *p*_*c*_(*i*) should be bigger to produce a greater number of better individuals; otherwise, it should be smaller because the evolving individuals are becoming better. The improved adaptive crossover operation from Eq (20) is

$$\alpha_{ik}^{\prime }=\left\{ \begin{array}{c} D_{best}(k,k),\;if\;rand(k)<p_{c}(i)\\ \alpha_{ik},\;otherwise \end{array}\right.,i=1,2,\ldots ,m;k=1,2,\ldots N,$$ (23)

A better population is determined after the crossover operation following the pseudocode.

start

        obtain fitness(i) in parallel;

        find f(max) and f(min);

        obtain pc(i) according to formula (22) in parallel;

        obtain new binary population;

end

where fitness(i) is the fitness value of the i^*th*^ individual.

### Rotation angle updating rules

Learning from the solid lookup rules [39], we present a set of adaptive rotation angle updating rules in Table 1. The rotation angle *θ*_*i*_ (*θ*_*i*_ = δ_*i*_ * s(*α*_*i*_,*β*_*i*_)), *i* = 1,2,…,*m* dynamically varies according to the evolution process.

**Table 1:** Rotation angle updating rules.

D_c_(i)	D_best_(i)	f(D_c_) < *f*(D_best_)	s(*α*_*i*_,*β*_*i*_)				
			δ_*i*_	*α*_*i*_*β*_*i*_>0	*α*_*i*_*β*_*i*_<0	*α*_*i*_ = 0	*β*_*i*_ = 0
0	0	false	0	0	0	0	0
0	0	true	0	0	0	0	0
0	1	false	$\frac{f(D_{c})}{f(D_{best})}∙0.03\pi$	+1	−1	0	±1
0	1	true	$\frac{f(D_{c})}{f(D_{best})}∙0.01\pi$	−1	+1	±1	0
1	0	false	$\frac{f(D_{c})}{f(D_{best})}∙0.03\pi$	−1	+1	±1	0
1	0	true	$\frac{f(D_{c})}{f(D_{best})}∙0.01\pi$	+1	−1	0	±1
1	1	false	0	0	0	0	0
1	1	true	0	0	0	0	0

If D_c_(i) ≠ D_best_(i), the rotation angle δ_*i*_ is adaptively proportional to $\frac{f(D_{c})}{f(D_{best})}$. If f(D_c_) < *f*(D_best_), the angle will be smaller; otherwise, it will be bigger. Initially, a big initial angle is set. As the iteration proceeds, the differences between individuals decrease and δ_*i*_ becomes smaller. In this way, the probability amplitude evolves in the direction of the optimal solution.

### Population catastrophe

When the best individuals in several successive generations are identical, it shows that the algorithm falls into a local minimum. At this moment, catastrophe operations for the current population should be performed to take it out of the constraint and start a new search. Specifically, the successive best individual is retained in the new population Q(t + 1) and the remaining individuals in Q(t + 1) are regenerated as a large disturbance. The pseudocode of the catastrophe operation is as follows:

start

        obtain the best individual corresponding to the optimal fitness;

        keep this best individual;

        rebuild the rest in parallel;

end

The strategy would prefer that the population eliminate its dull state rather than make it degenerate, which is an effective means to commence a new search.

## Simulations and analyses

**Fig 8:**
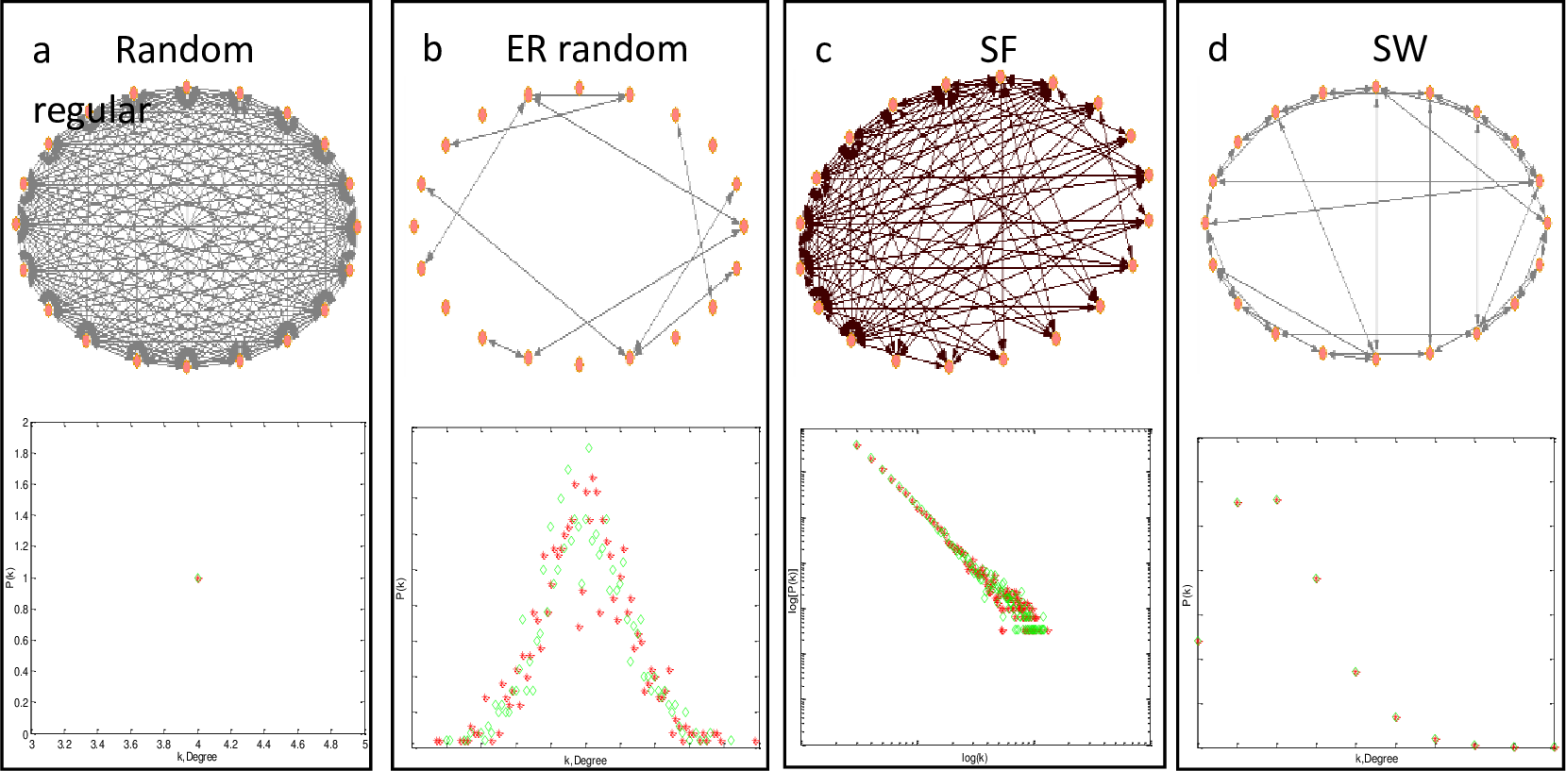
Characteristics of the addressed networks. Red stars represent the node in-degree denoted by ⟨k_in_⟩ and the green diamonds represent the node out-degree denoted by ⟨k_out_⟩. (a) Random regular networks with homogeneous degree distribution of ⟨k_in_⟩ = ⟨k_out_⟩ = 4. (b) ER random networks with Poisson degree distribution; the degree heterogeneities rely on the average degree denoted by ⟨k⟩. (c) SF networks with power-law degree distribution, which results in large degree heterogeneities. (d) SW networks with long-tail degree distribution, which decreases much slower than the SF distribution.

We used the orthodox ER random [46], SF [47], SW networks of NW type [48] and some real-world networks as benchmarks to illustrate the feasibility of the PAQGA for optimizing the controllability of arbitrary networks that encompass control nodes and state nodes. Additionally, we also conducted an analysis of the relationship between the network topology and number of control nodes. ER and SF networks were obtained from the static model [49] with *N* state nodes and *P* candidate control nodes (N = *P*). Each control node pointed to state nodes with uniform probability and the weights of all edges were randomized between zero and one. SW networks were generated from randomized adding edges [48, 49]. The characteristics of random regular networks, ER, SF, and SW networks are illustrated in Fig 8.

We define the number of selected control nodes that correspond to the current best control scheme as n_*c*_ and the density of these selected control nodes as N_*c*_, where N_*c*_ = n_*c*_/*N*. The minimum number of selected control nodes after the optimization process is denoted as n_*cm*_, and the minimum control node density is N_*cm*_ = n_*cm*_/*N*. To implement the parallel strategy, we performed the following simulations on eight MATLAB^®^ workers. The parameters of the PAQGA were set to 2m = 30, maxgen = 100, and *p*_*c*0_ = 0.06. All the following experimental results are the average of 10 independent simulations and the standard deviation is 0.01.

### Performance of the PAQGA

**Fig 9:**
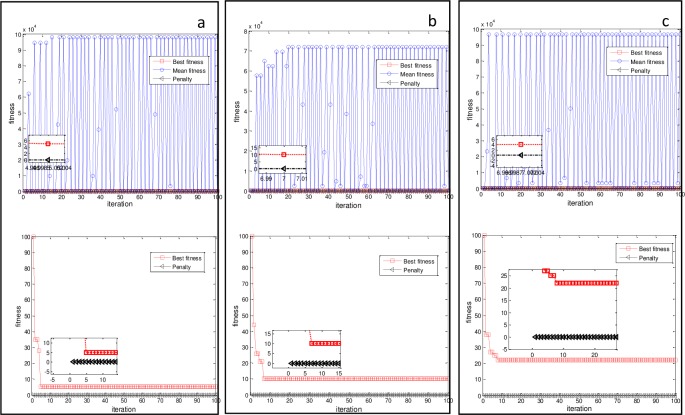
Fitness and penalty curves as a function of iterating generation for (a) ER with ⟨k⟩ = 4.0, (b) SF with ⟨k⟩ = 4.0 and γ = 2.1, and (c) SW with ⟨k⟩ = 4.0. The red dotted line with a square is the best fitness corresponding to D_best_ at the current generation, the blue dashed line with a circle is the mean fitness of all control schemes at each generation, and the black line with a triangle is the penalty term corresponding to D_best_ at each generation.

To show that the optimal solution (*D*_*best*_) at each generation always evolves in the feasible region, that is, *Pen*_*i*_(*D*_*best*_) = 0, we conducted experiments on different networks. All these networks were directed with 100 state nodes and 100 candidate control nodes. The experimental results are shown in Fig 9. The figure shows that the best fitness quickly converged to the minimum value after approximately the first few generations. The mean current fitness fluctuated dramatically because of the operations of qubit cross, qubit catastrophe, and Q-gate rotation. The penalty was always equal to zero, which means that *D*_*best*_ always met the PBH rank condition throughout the entire optimization process.

We compare the performance of PAQGA of optimizing network controllability with that of EO [22] and adaptive GA [28] on a list of popular networks and real-life networks. The comparison results are tabulated in Table 2.

**Table 2:** Performance comparison of PAQGA, GA, and EO on different networks in terms of *n*_*cm*_, the minimum iterating generations, and computational time. Power-law index of SF networks in these experiments was γ = 2.1. ‘/’ indicates that the corresponding results were not available for the computational time limit. For data sources, see Supplementary information S1 Dataset.

network	N/P	<k>	PAQGA			Adaptive GA [28]			EO [22]		
			ncm	iterations	time (s)	ncm	iterations	time (s)	ncm	iterations	time (s)
ER	25	1.5	2	3	1.86	2	30	0.23	3	22	0.45
ER	50	3	3	4	4.03	3	34	4.19	3	22	7.79
ER	100	4	5	5	7.38	5	75	266.26	6	24	121.62
ER	200	6	31	5	12.49	31	128	873.05	31	29	1545.84
ER	300	8	22	11	18.31	25	44	1708.81	/	/	/
ER	500	10	43	17	31.82	/	/	/	/	/	/
ER	1000	16	64	26	65.05	/	/	/	/	/	/
SF	25	1.5	3	5	2.36	3	20	0.43	4	22	0.75
SF	50	3	5	6	5.38	5	32	4.83	5	21	8.37
SF	100	4	10	7	8.22	10	69	278.55	10	21	134.54
SF	200	6	35	5	13.06	35	116	892.16	36	34	1623.13
SF	300	8	83	11	19.57	83	132	1823.12	/	/	/
SF	500	10	192	17	34.92	/	/	/	/	/	/
SF	1000	16	416	26	68.04	/	/	/	/	/	/
Rhode [50]	20	2.65	2	5	2.76	2	20	0.52	2	22	0.86
Maspalomas [50]	24	3.417	3	6	5.56	3	32	5.24	3	21	8.93
Michigan [50]	39	5.667	13	7	6.45	13	69	5.52	14	21	12.62
Circuit-s208 [51]	122	3.126	29	9	18.22	29	116	913.14	30	34	1745.83
Friend-rev [52]	228	4.01	52	10	20.45	52	121	1201.54	54	45	2733.61
Circuit-s420 [51]	252	3.21	59	13	23.65	59	132	1962.92	/	/	/
Circuit-s838 [51]	512	3.44	119	18	39.03	/	/	/	/	/	/
Roget [50]	1022	4.966	396	27	75.66	/	/	/	/	/	/

From the columns of *n*_*cm*_, it can be observed that the three algorithms almost converged to the same value, which demonstrates that PAQGA, GA, and EO all had a good ability to determine the optimal control nodes. When the size of the network was small (e.g., N = P ≤ 50), PAQGA took slightly more time than GA and EO to determine the best solution. This paradoxical phenomenon is attributed to the launching of the MATLAB^®^ distributed server, and the launching time was approximately 2s. However, once the server started, PAQGA showed a greater advantage in processing large-size networks over GA and EO. For example, for the ER network with ⟨*k*⟩ = 6.0 and N = P = 200, PAQGA obtained *n*_*cm*_ at the fifth generation and took 12.49s; for the same network, GA took 873.05s at the 128^th^ generation and, and EO required 29 generations and 1545.84 s. By comparing the computational time, PAQGA saved 98.57% more than GA and 99.19% more than EO.

A parallel version of GA was transformed from the adaptive GA [28] using *η* MATLAB® workers with the computational complexity of $O(\frac{2m\times {\left(N+P\right)}^{3}\times l}{\eta })$, where 2m is the population size, *l* is the number of different eigenvalues of the controlled network. We compared it with the proposed PAQGA, and the results are shown in Table 3.

**Table 3:** Performance comparison of PAQGA and parallel GA on different networks using eight MATLAB® workers in terms of n_cm_, the minimum iterating generations, and computational time. For data sources, see Supplementary information S1 Dataset.

network	N/P	<k>	PAQGA			parallel GA		
			ncm	iterations	time (s)	ncm	iterations	time (s)
ER	25	1.5	2	3	1.86	2	19	2.71
ER	50	3	3	4	4.03	3	28	5.19
ER	100	4	5	5	7.38	5	35	53.31
ER	300	8	22	11	18.31	25	37	89.54
SF	25	1.5	3	5	2.36	3	18	3.52
SF	50	3	5	6	5.38	5	27	6.65
SF	100	4	10	7	8.22	10	34	42.73
SF	300	8	83	11	19.57	83	126	243.12
Rhode [50]	20	2.65	2	5	2.76	2	18	3.51
Maspalomas [50]	24	3.417	3	6	5.56	3	28	8.32
Michigan [50]	39	5.667	13	7	6.45	13	66	11.47
Circuit-s208 [51]	122	3.126	29	9	18.22	29	98	220.78
Friend-rev [52]	228	4.01	52	10	20.45	52	107	275.67
Circuit-s420 [51]	252	3.21	59	13	23.65	59	116	295.63
Circuit-s838 [51]	512	3.44	119	18	39.03	119	122	413.41
Roget [50]	1022	4.966	396	27	75.66	396	135	511.76

It can be inferred from Table 3 that the parallel computation (allowing for multiple processors) contributes to the performance of algorithms. However, it is not the only important factor. The computational efficiency of EO, GA, parallel GA and PAQGA could be reflected by their computation complexity. First, the computation of the PBH rank matrix in GA, parallel GA and PAGQA and the Kalman rank matrix in EO is the most time-consuming. This is the main factor affecting their speedability. The rank computation of Kalman matrix takes much more time than that of PBH matrix. Second, PAQGA adopts qubits representation, where each chromosome contains two individuals. This expands the space of feasible solution. And the adaptive Q-gate rotation operation and crossover operation help to improve the algorithm efficiency.

Moreover, comparison tests among the PAQGA, MMT and MM are conducted to observe the performance of the PAQGA on much larger real networks. The experimental results are shown in Table 4.

**Table 4:** Performance comparison of PAQGA, MMT, and MM on several large real-directed, -weighted and–unweighted networks in terms of N_cm_ and computational time. For data sources, see Supplementary information S1 Dataset.

network	class	N/P	PAQGA		MMT [11]		MM [10]	
			Ncm	time (s)	Ncm	time (s)	Ncm	time (s)
Coauthorships [53]	Directed weighted	1461	0.3436	85.12	0.3436	67.03	0.3425	34.16
SciMet [54]	Directed unweighted	2729	0.4251	126.35	0.4251	83.29	0.4236	52.91
Kohonen [54]	Directed unweighted	3772	0.562	173.46	0.562	106.62	0.5604	73.85
Wiki-Vote [55]	Directed unweighted	7115	0.6656	392.44	0.6656	228.73	0.6656	167.59
P2P-3 [56]	Directed unweighted	8717	0.5778	473.19	0.5778	279.15	0.5774	206.52
P2P-1 [56]	Directed unweighted	10876	0.5531	685.78	0.5531	359.83	0.552	268.96

It can be seen that N_*cm*_ of PAQGA agrees with that of MMT on these real-world networks, and is slightly greater than or equal to that of MM. The experimental results show the efficiency of PAQGA in identifying the minimum control nodes. Nevertheless, PAQGA is at a disadvantage in computational time compared with MMT and MM, although such defect could be improved by adding the number of processors or using computer groups. For example, the cost of calculating N_*cm*_ of Wiki-vote network is reduced to 301.34s with 12 processors.

**Fig 10:**
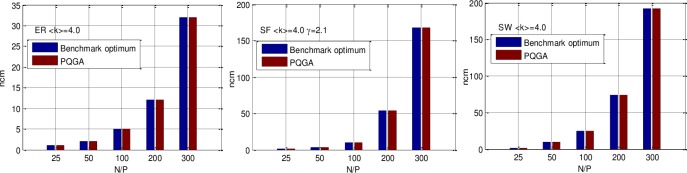
n_cm_ comparison between the structural controllability theory and PAQGA on (a) ER with ⟨k⟩ = 4.0, (b) SF with ⟨k⟩ = 4.0 and γ = 2.1, and (c) SW with ⟨k⟩ = 4.0.

PAQGA is an intelligent probabilistic optimization algorithm that provides approximate solutions. The optimal solution cannot be guaranteed to be found. Moreover, the optimal solution of a problem is typically unknown in advance. We used the structural controllability theory [10, 18] as the benchmark to compute *n*_*cm*_ to test the validation of PAQGA. The results are shown in Fig 10.

We can observe that for ER, SF, and SW, the obtained *n*_*cm*_ is the same as the benchmark result, which indicates that the proposed PAQGA was effective in determining the minimum control nodes of complex networks.

### Discussion and analysis of results

**Fig 11:**
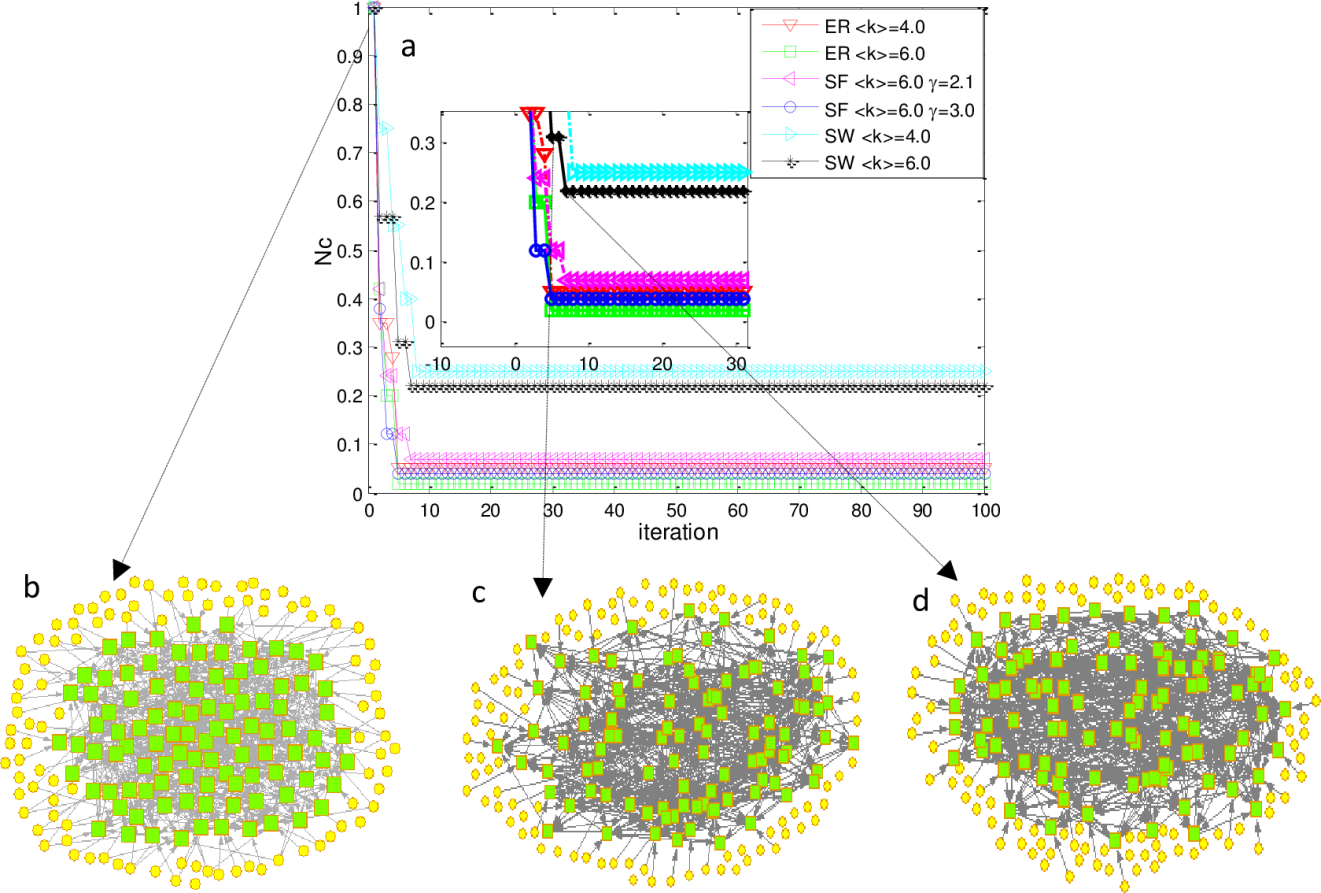
PAQGA optimization results and network topology evolution. (a) Convergence trend of N_c_ of directed ER, SF, and SW with N = P = 100. (b) Initial network topology (at the zeroth generation) of SW with ⟨k⟩ = 6.0. Yellow circles represent the candidate control nodes and green squares represent the state nodes. Selected control nodes are connected to state nodes with a row from circles to squares. Links between state nodes are arrowed lines between squares. (c) Network topology guided by 31 control nodes at the fifth generation. (d) Network topology with 22 control nodes at the first convergence generation (seventh generation).

Applying PAQGA, the optimization results and evolution process of network topology can be achieved. The results are intuitively displayed in Fig 11.

From Fig 11(A), N_c_ of different networks quickly converged to a steady minimum value, which indicates the effectiveness of the PAQGA. For example, *N*_*c*_ of ER with <k> = 4.0 converged to 0.05 at the fifth generation, which demonstrates that five control nodes were sufficient to maintain network controllability. For SF with <k> = 6.0 and γ = 2.1, *N*_*c*_ rapidly decreased to a minimum value of 0.07 at the seventh generation, which means that at least seven control nodes were required to fully control the network. Fig 11(B)–11(D) together capture the evolution of the SW network with <k> = 6.0 at the zeroth, fifth, and seventh iteration, and the convergence trend of the control nodes can be acquired.

We also found that two networks with different <k> required different *N*_*cm*_. For example, for ER with <k> = 4.0 and <k> = 6.0, *N*_*cm*_ of the network with <k> = 4.0 was 0.05 and with <k> = 6.0, *N*_*cm*_ = 0.02. Additionally, *N*_*cm*_ of SW networks with <k> = 4.0 and <k> = 6.0 was 0.25 and 0.22, respectively. Second, for networks with the same <k> and different γ, *N*_*cm*_ also differed. For example, consider SF with same <k> = 6.0, and different γ = 2.1 and γ = 3.0. The two networks had *N*_*cm*_ = 0.06 and *N*_*cm*_ = 0.04, respectively. Third, for networks with the same γ and different <k>, N_cm_ was also different, which can be determined from Table 2. These results led us to conjecture that *N*_*cm*_ had a relationship with <k> and γ.

**Fig 12:**
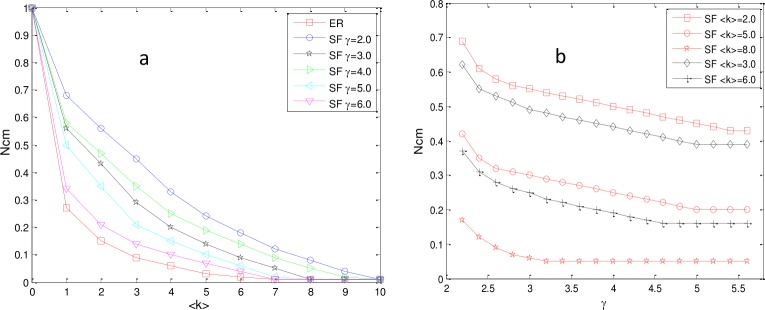
Impact of <k> and γ on *N*_*cm*_. (a) N_cm_ as a function of <k> with fixed γ. (b) *N*_*cm*_ as a function of γ with fixed <k>. Networks are directed with N = P = 500.

To confirm our hypothesis, we performed simulations on a set of different networks and plotted *N*_*cm*_ as the function of <k> and γ. The results are shown in Fig 12.

From Fig 12(A), it is obvious that *N*_*cm*_ of networks with fixed γ decreased monotonically with <k> until *N*_*cm*_ became slowly flat. Additionally, the downward trend was of asymptotic exponential dependence, which suggests that the sparse network required more control nodes to maintain full controllability. From Fig 12(B), we can observe that N_cm_ with fixed <k> decreased as γ increased. The results indicate that N_cm_ may be influenced by the degree heterogeneity, denoted by H, which is the standard deviation of the network node degree distribution [57]. In this paper, H is defined as

$$H={\left(\sum_{i}\left(k_{i}-{\left\langle k\right\rangle )}^{2}\right)/N\right)}^{1/2},\;i=1,2,\ldots ,N,$$ (24)

where k_i_ is the degree of state node i.

**Fig 13:**
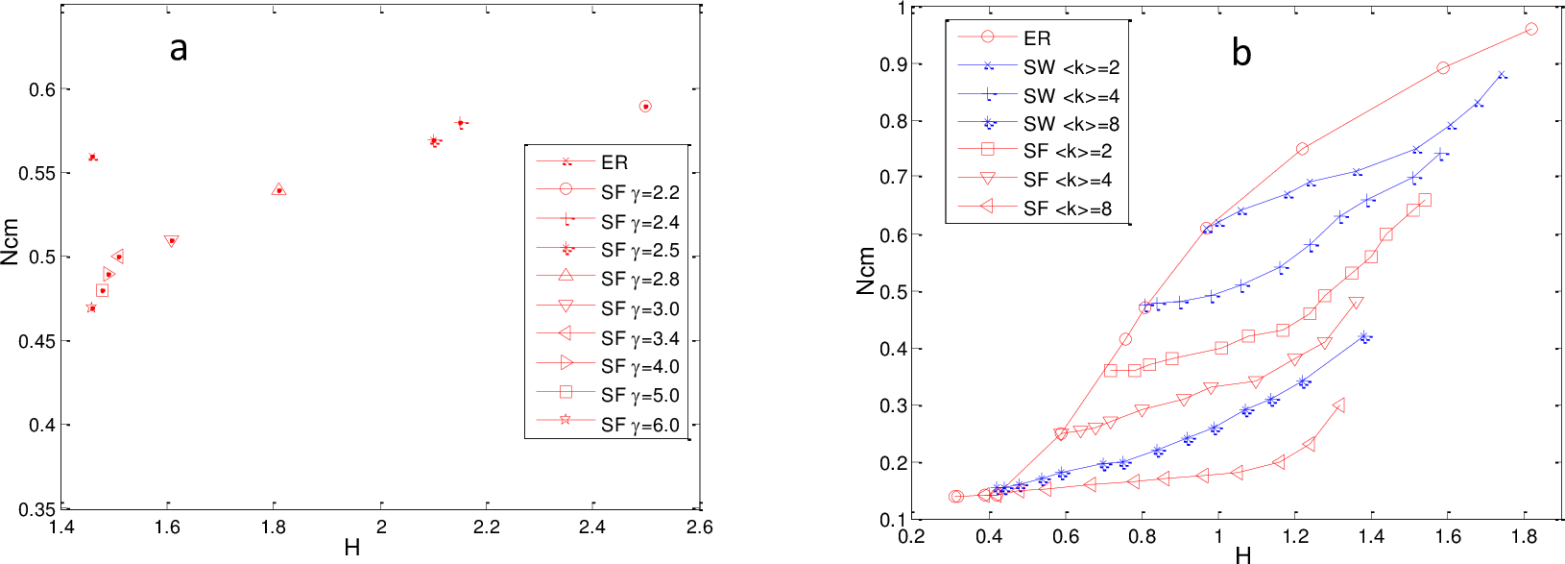
N_cm_ as a function of H. (a) N_cm_ as a function of H for ER and SF networks with fixed γ and variable ⟨k⟩. (b) N_cm_ as a function of H for ER, SF, and SW networks with variable γ and fixed ⟨k⟩. The networks are directed with N = P = 500.

To determine the relationship between N_cm_ and H, we examined N_cm_ as a function of H and obtained the results shown in Fig 13.

From Fig 13(A), it can be observed that a larger N_cm_ always corresponded to a larger H and smaller γ. Fig 13(B) shows that the network with a smaller ⟨k⟩ and larger H typically required a larger N_cm_. The results suggest that the larger the differences between node degrees, the more control nodes were required to entirely control the network.

**Fig 14:**
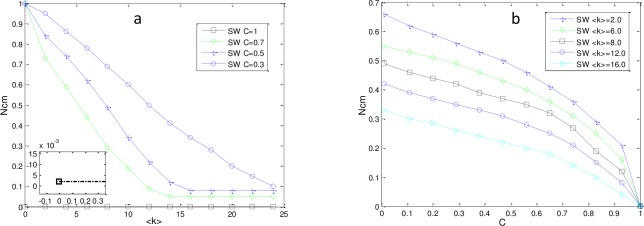
Impact of <k> and C on N_cm_ of SW networks. (a) N_cm_ as a function of <k> with fixed C. When C = 1, the network is fully connected and can be steered to any state with only one controller. (b) N_cm_ as a function of C with fixed ⟨k⟩. Networks are directed with N = P = 500.

SW networks have the remarkable characteristics of a large clustering coefficient, denoted by C, which represents the overlapping degree of friend circles of two adjacent state nodes and is defined as

$$C=\frac{1}{N}\sum_{i}\frac{E_{i}}{\frac{1}{2}k_{i}(k_{i}-1)},\;i=1,2,\ldots ,N,$$ (25)

where i is node i, k_i_ is the number of edges between node i and other nodes, and E_i_ is the number of edges among the k_i_ nodes. For SW networks, N_cm_ may be affected by C. To explore the relationship between N_cm_ and C, we plot N_cm_ as a function of ⟨k⟩ and C,shown in Fig 14.

From Fig 14(A) and 14(B), we can determine that a larger C corresponded to a smaller N_cm_, which indicates that the more interconnected the network, the fewer control nodes were required to control the network. For other networks, such as ER, SF, the conclusion also holds.

Considering the aforementioned analysis results together, we can determine that for a given network with both control nodes and state nodes: 1) the sparser the network, the more control nodes were required to control it; and 2) the more heterogeneous the network, the more control nodes were required to guarantee its full control. We reflect that the sparse and heterogeneous network is the most difficult for guiding its dynamic evolution (see Tables 2 and 4 and Figs 10(A) and 12(B)). The consistency between the results from our approach and from these existing methods [10, 11, 22, 28] confirms the similarity between them for directed networks, which not only further validate these existing methods, but also reflect the effectiveness of our method.

To evaluate the controllability of directed networks, the structural controllability framework based on the MM method is still the best for its error-free feature [11]. Like the MMT, the PAQGA also relies on the eigenvalues and the rank of the network matrix, the computation of which inevitably introduces numerical errors. Further, MM and MMT both surpass PAQGA in computational efficiency in identifying both the minimum set of driver nodes and the number of these driver nodes. However, the PAQGA can have a wider range of applications. For example, the PAQGA is valid for networks containing a number of self-loops with identical or different weights, and networks with bidirectional connections between two nodes. The PAQGA is also applicable to undirected networks, where the structural matrix assumption is slightly violated because of the network symmetry. Further, combined with advantages of computer hardware and the adaptive strategies itself, PAQGA has great room for improvement. Taken together, the PAQGA as an alternative exact structural controllability framework provides us deeper understanding of the controllability of complex networked systems.

## Conclusions

In this paper, we introduced a PAQGA to optimize the controllability of arbitrary networks with control nodes and state nodes under the PBH rank condition. In addition to MATLAB® workers, more parallel mechanisms can be flexibly embedded in the PAQGA, for which more threads concurrently processing could further promote the time efficiency of generating a solution. Analyses and simulation comparisons demonstrated the effectiveness and applicability of the proposed PAQGA. Furthermore, we found that the minimum control nodes were affected by the network degree distribution, degree heterogeneity, and clustering coefficient. The sparse and heterogeneous network is the most difficult to be fully controlled.

In our study, the topology that comprises state nodes remained static during the entire evolution process. However, networks normally evolve over time, which manifests as the increasing or decreasing of different nodes and their links. In the future, we will focus on the controllability of dynamic networks. Furthermore, we hope to explore how to use the obtained minimum control nodes to steer an intermediate network to evolve into our desired network considering realistic energy constraints.

## Supporting information

S1 Dataset Canonical and real-world network datasets for comparison experiments. (RAR)
